# The Development and Validation of Models of Risk for Behaviours That Challenge in Children With Developmental Disabilities: A Novel Machine Learning Approach

**DOI:** 10.1111/jir.70105

**Published:** 2026-04-21

**Authors:** Laura Groves, Guy Davies, Chris Oliver, Debbie Allen, Charlie Bamford, Louise Bell, Chloe Brown, Vivian Cooper, Louise Daniel, Jo Garstang, Chris Jones, Joseph P. McCleery, Ashley Liew, John Rose, Doug Simkiss, Catherine Steenfeldt‐Kristensen, Alice Welham, Caroline Richards

**Affiliations:** ^1^ School of Psychology University of Birmingham Birmingham UK; ^2^ Birmingham and Solihull Mental Health NHS Foundation Trust Birmingham UK; ^3^ School of Physics and Astronomy University of Birmingham Birmingham UK; ^4^ School of Education & Psychology in the Faculty of Education, Health, & Wellbeing University of Wolverhampton Wolverhampton UK; ^5^ Wolverhampton and Sandwell CAMHS Black Country Healthcare NHS Foundation Trust Wolverhampton UK; ^6^ Sefton Community Learning Disabilities Team Mersey Care NHS Foundation Trust Southport UK; ^7^ Adult Learning Disabilities Community Health Team South West Yorkshire Partnership NHS Foundation Trust Wakefield UK; ^8^ The Challenging Behaviour Foundation Chatham UK; ^9^ Specialist Assessment Service, University Hospitals Birmingham NHS Foundation Trust Chelmsley Wood Primary Care Centre Birmingham UK; ^10^ Birmingham Community Healthcare NHS Foundation Trust; Birmingham School of Nursing University of Birmingham Birmingham UK; ^11^ Department of Psychology & Kinney Center for Autism Education and Support Saint Joseph's University Philadelphia Pennsylvania USA; ^12^ Center for Autism Research Children's Hospital of Philadelphia Philadelphia Pennsylvania USA; ^13^ National & Specialist CAMHS South London & Maudsley NHS Foundation Trust London UK; ^14^ Paediatric Neurosciences Evelina London Children's Hospital London UK; ^15^ Institute for Mental Health University of Birmingham Birmingham UK; ^16^ Children's Neurodevelopmental Service Coventry and Warwickshire Partnership Trust Coventry UK

## Abstract

**Background:**

Children with developmental disabilities show a high prevalence of behaviours that challenge (BtC). Thus, harnessing known risk markers to target early intervention to children at the greatest risk of BtC is essential. In this study, machine learning techniques were used to develop prediction models of risk (no, low and high severity behaviour) for different BtC (self‐injurious behaviour, aggression, property destruction, ‘any BtC’). A secondary aim was to assess the external validation of these models to predict future behaviour.

**Method:**

Caregivers of individuals with developmental disabilities completed the Self‐injury, Aggression and Destruction Screening Questionnaire. One dataset (*n* = 778) was used to train and test models to establish internal validation. Algorithms were created using random forest classifiers, K‐nearest neighbours, multiple logistic regressions and Gaussian mixture models (GMM) for each type of behaviour. External validation utilising a second dataset of caregivers (*n* = 121) completing the SAD‐SQ at baseline and 12 months later was then conducted.

**Outcomes:**

Across internal and external validation, the random forest classifiers and GMM algorithms for *any BtC* showed the highest number of correct classifications with fair to good recall and precision, with 83.5% of people at risk of BtC correctly predicted. Predictions of persistence and incidence of behaviour over 12 months was also good (83.5% and 83.3%, respectively).

**Interpretation:**

The novel prediction models showed the ability to predict BtC for children with developmental disabilities. Such models have applicability to clinical practice to inform provision of early preventative interventions for BtC.

## 1 Introduction

Approximately 1% of individuals have a developmental disability (DD; McKenzie et al. 2016) with 10%–15% of this population showing behaviours of concern (BtC; Cooper et al. 2009; Emerson et al. 2001). These behaviours are often chronic, persistent and associated with lower quality of life (Beadle‐Brown et al. 2009; Laverty et al. 2020; Richards et al. 2016). Within the broad category of BtC, self‐injury, aggression and property destruction are the most concerning to families, healthcare, education and care staff (Lowe and Felce 1995a, 1995b). These BtC are associated with increased physical restraint and hospitalisation, off‐label medication use, polypharmacy, greater parental stress, staff burnout and increased financial cost to the healthcare system (Adams et al. 2016; Allen et al. 2009; Knapp et al. 2005; McMahon et al. 2020). Thus, it is critical that early, preventative interventions are available to those at risk of developing BtC (Banks and Bush 2016; National Institute for Health and Care Excellence [NICE] 2015).

Previous research has identified risk markers for BtC including chronological age, level of ability, presence of autism characteristics, physical health conditions, repetitive behaviour, impulsivity and overactivity (Dimian and Symons 2022; McClintock et al. 2003; Oliver and Richards 2015). National guidance highlights that these risk markers should be used to inform the provision and delivery of early intervention; however, access to services remains inconsistent with long waitlists (NICE 2015; Beresford 1995; Redmond and Richardson 2003; Ruddick et al. 2015). Consequently, children commonly only gain access to support once behaviour has reached a crisis point that is associated with poorer long‐term outcomes (Davies and Oliver 2016; Emerson et al. 2001; Laverty et al. 2020; Richards et al. 2016).

One reason for the limited provision of early support for BtC is a lack of acknowledgement of chronicity and the limited translation of data on risk markers to organise and structure service provision effectively. Drawing on stepped care approaches, early support services could be stratified based on risk for the presence and severity of BtC. In such a model, greater resources would be allocated to those at risk of showing high severity BtC, followed by those individuals at risk of low severity BtC. Existing examples of early screening and risk detection to stratify service delivery in general healthcare include cervical screenings and the NHS health checks where early biological indicators of disease are investigated (NHS Digital 2016; Public Health England 2019).

To move towards this efficient model of service delivery for BtC, an appropriate screening tool that assesses known predictors of BtC and provides risk categorisation is required. An appropriate and effective screening measure to assess risk of BtC should be short and easy for caregivers or clinicians to complete within clinical settings. It must also be appropriate for use in a child's care pathway where they may not yet be exhibiting or seeking support for BtC, in order to facilitate a preventative care model. The Self‐injury, Aggression and Destruction Screening Questionnaire (SAD‐SQ) is one such promising candidate (Davies and Oliver 2016). However, to inform a stratified delivery of services, the outcomes of such a screening measure are required to provide statistical classifications of level of risk.

Machine learning models are frequently used in medical research to make classifications of risk of disease utilising known risk markers (Krittanawong et al. 2020; Qiu et al. 2020; Khanam and Foo 2021; Kotsiantis 2007). These approaches have been applied to the behavioural sciences to predict diagnosis and model risk of mental health conditions such as anxiety, depression and suicide attempts, as well as BtC (Plötz et al. 2012; Tran and Kavuluru 2017; Priya et al. 2020; Walsh et al. 2017). Machine learning has also been used to predict risk of first episode psychosis and may inform the delivery of preventative interventions in this population (Leighton et al. 2019; Fusar‐Poli et al. 2020). Such studies demonstrate the feasibility and applicability of machine learning to model and predict complex behaviour. However, no previous study has applied machine learning approaches to predict classifications of BtC to specifically inform service delivery.

In this study, machine learning approaches were used to develop prediction models of risk of BtC severity with a large sample of individuals with DD. The performance of these prediction models is assessed in this sample and in an additional community sample of children with longitudinal data (T1: baseline; T2: 1‐year follow‐up). This approach allowed exploration of the generalisability of these prediction models to an external dataset, as well as the ability of the models to detect incidence, persistence and remission of BtC over time.

### 1.1 Aims

The aims of this study are:

1. To use machine learning approaches to develop prospective predictive models of the risk of children showing no, low or high severity BtC.
2. To evaluate the performance of these models with specific attention to their predictive ability to identify (a) presence of BtC and (b) severity of BtC in community samples.
3. To assess the accuracy of these models to predict change in BtC over time in community samples and determine the utility of such models within clinical settings.
4. To determine the contribution of individual predictor variables in the development of predictive models of risk

## 2 Methodology

### 2.1 Participants

Two datasets are included in this study forming an internal validation dataset and an external validation dataset.

#### 2.1.1 Internal Validation Dataset

The internal validation dataset comprised 778 parents/carers of children with DD aged 1‐15 years who participated in previous research (Davies and Oliver 2016; Handley 2014, ^1^; Richards et al. 2017) Participants were recruited via Child Development Centres (*n* = 280, 36.0%) and Special Needs schools (*n* = 498, 64.0%).

#### 2.1.2 External Validation Dataset

The external validation dataset comprised 121 parents/carers of children with a confirmed or suspected DD aged 2–11 years. Participants were recruited via Child Development Centres (*n* = 59, 48.8%), Special Needs Schools (*n* = 32, 26.4%) and a database of families with children with genetic syndromes associated with DD held by the Cerebra Network for Neurodevelopmental Disorders (*n* = 30, 25.8%).

Child predictor variables and BtC outcomes are presented in Tables 1 and 2. Between‐group analyses of predictor variables and BtC outcomes revealed significant differences between the datasets across all variables (*p* < 0.05; external validation using T1), with the exception of sex, adaptive behaviour and autism characteristics. The heterogeneity between the development and external validation datasets is expected given the population under assessment. Such variability between the samples affords an opportunity to determine how well prediction models generalise to a novel, distinct dataset with unique characteristics.

**TABLE 1:** Predictor variables for internal and external validation datasets.

		Internal validation dataset (*n* = 778)	External validation dataset^a^ (*n* = 121)	ꭓ^2^/U	df	*p*
Chronological age (years)	Median (IQR)	8.00 (5.00–10.00)	7.00 (5.00–9.00)	41545.00		**0** **.** **037** *
Sex	*N* (%) male	574 (73.80)	83 (67.80)	1.918	1	0.166
Adaptive behaviour	*N* (%) low ability	416 (53.50)	71 (58.70)	1.144	1	0.285
Autism	*N* (%) present	480 (61.70)	75 (62.00)	0.004	1	0.952
Number of health problems	Median (IQR)	1.00 (0.00–2.00)	2.00 (1.00–3.00)	30124.00		**< 0.001** **
Repetitive behaviour		1.00 (1.00–3.00)	2.00 (1.00–4.00)	41695.00		**0** **.** **039** *
Obsessive behaviour		2.00 (1.00–4.00)	3.00 (1.00–4.00)	41241.00		**0** **.** **025** *
Impulsivity		4.00 (1.00–6.00)	6.00 (3.00–8.00)	32367.00		**< 0.001** **
Overactivity		2.00 (0.00–5.25)	4.00 (2.00–6.00)	36731.00		**< 0.001** **

^a^ Predictor variables based on T1 data.

* *p* < 0.05.

** *p* < 0.001.

**TABLE 2:** BtC outcomes for internal and external validation datasets.

		Internal validation dataset (*n* = 778)	External validation dataset (*n* = 121)		Between‐group comparisons		
			*T1*	*T2*			
		**A**	**B**	**C**	**A** versus **B**	**A** versus **C**	**B** versus **C**
Self‐injurious behaviour	Severity of behaviour (*n*): No, low, high	542, 158, 74	53, 49, 19	47, 55, 19	ꭓ^2^ = 32.870, ***p* < 0.001** **	ꭓ^2^ = 46.831, ***p* < 0.001** **	*p* = 0.407^a^
Aggression		340, 320, 106	27, 60, 34	26, 63, 32	ꭓ^2^ = 27.119, ***p* < 0.001** **	ꭓ^2^ = 26.378, ***p* < 0.001** **	*p* = 0.885^a^
Destruction of property		475, 211, 85	46, 51, 24	40, 60, 21	ꭓ^2^ = 24.389, ***p* < 0.001** **	ꭓ^2^ = 35.289, ***p* < 0.001** **	*p* = 0.132^a^
Any BtC		294, 328, 156	17, 60, 44	18, 61, 42	ꭓ^2^ = 30.922, ***p* < 0.001** **	ꭓ^2^ = 27.707, ***p* < 0.001** **	*p* = 0.790^a^

^a^ McNemar test.

** *p* < 0.001.

### 2.2 Measures

The SAD‐SQ (Davies and Oliver 2016) was selected as the primary measure for this study.

#### 2.2.1 Choice of Primary Measure

The SAD‐SQ is a short questionnaire assessing a broad range of BoC. Measurement of risk markers (chronological age, adaptive behaviour/level of ability, autism characteristics, health problems, overactivity, impulsivity and repetitive behaviour) was also elicited through items in the SAD‐SQ (Davies and Oliver 2016) with item description and value range presented in Table S1. Inter‐rater reliability for the SAD‐SQ is fair to good, with strong concurrent and convergent validity (Davies and Oliver 2016). Preliminary evidence also suggests that the SAD‐SQ is able to predict incidence of BtC over time (Davies and Oliver 2016). The SAD‐SQ was selected for its balance between collecting a wide range of information with brevity and ease of completion by parents and clinicians. This makes this measure a promising candidate for an effective screening measure for BtC.

The SAD‐SQ assesses frequency of BtC on a Likert scale from 0 to 4 with higher scores indicating more frequent and severe behaviour. For this study, individuals with a score of 0 indicating no behaviour was present formed the *no behaviour* group. Individuals with a score of 4 representing the most frequent and severe behaviour formed the *high severity behaviour* group. Finally, individuals with a score of 1, 2 or 3 indicating presence of behaviour with comparatively lower frequency and severity formed the *low severity behaviour* group. These groupings meant that the model performances could be assessed in two ways with a view toward translation to a risk‐informed service model: firstly, the ability of the models to identify whether a child required any preventive intervention (*low and high severity* behaviour compared to *no behaviour* group) and, secondly, to evaluate effective stratification between the *low* and *high severity* behaviour groups.

### 2.3 Procedure

For the internal validation dataset, data from participants who had taken part in previous research were pooled (Davies and Oliver 2016; Handley 2014; Richards et al. 2017). For the external validation dataset, prospective participants with children with confirmed or suspected developmental disabilities were provided with a covering letter of invitation, an information sheet, consent form and the SAD‐SQ at T1 (baseline). All participants who participated at T1 were followed up 12 months later to complete a second SAD‐SQ (T2; attrition = 54.5%).

### 2.4 Data Analysis

To address the first aim, prediction models for self‐injury, aggression, property destruction and any BtC were created from the internal validation dataset using four approaches: multinominal logistic regression, K‐nearest neighbours, Gaussian mixture models (GMMs) and random forest classifier. For all approaches, the internal validation dataset was divided into a training and test sample using a random 70:30 split. The training data were used to generate the models, and the test data were used to internally validate these.

#### 2.4.1 Internal Validation

To address the second aim, internal validation of the models was conducted using bootstrapping with 1000 replicates (Harrell 2006; Miao et al. 2013). Models were generated on the bootstrap replicates and applied to a non‐bootstrapped data set providing an ‘out of bag’ performance that was averaged.

#### 2.4.2 External Validation

To address the third aim, the performance of the final predictive models was evaluated on the longitudinal external validation dataset. Predictions of risk were calculated based on T1 data with presence and severity of behaviour at T1 and T2 serving as outcome variables. In addition, descriptive statistics of persistence, incidence and remission of BtC were calculated and compared to predicted risk categories to determine how effective models were at predicting dynamic changes over time.

#### 2.4.3 Performance Metrics

The models were evaluated by calculating balanced accuracy, precision, recall and area under the curve (AUC). Separate recall scores were also calculated to evaluate the correct classification of presence of behaviour (i.e., *no behaviour* vs. a composite *low and high severity* group) and the correct classification of *level* of behaviour severity (i.e., *low severity* vs. *high severity*). Finally, confusion matrixes were generated to aid evaluation and interpretation.

#### 2.4.4 Predictor Importance

To address the fourth aim, statistics describing how predictor variables contributed to the development of the models from the internal validation dataset were generated. For the multinominal logistic regressions, the coefficients of each predictor variable and associated significance were generated. For the random forest classifiers, the Gini impurity indexes were generated for each predictor variable. Due to how the KNN and GMM approaches classify cases, it was not possible to generate predictor importance for these analyses.

## 3 Results

### 3.1 Internal Validation

Performance metrics and confusion matrixes for all models are presented in Tables S2 and S3. The AUCs for all models ranged from 0.696 to 0.782, indicating good predictability. However, balanced accuracy across behaviour types showed the average proportion of correct classifications was comparatively low for self‐injurious behaviour (37.25%–52.73%) with property destruction (47.25%–58.50%), aggression (41.82%–59.72%) and any BtC performing slightly better (54.58%–59.93%). Across all models, recall showed that the correct classification of people with *no*, *low* or *high severity behaviour* ranged from 55.94% to 67.85%. Of the children classified by the model as showing *no*, *low* or *high severity behaviour*, between 56.24% and 67.53% of these were correct (precision).

The confusion matrixes (see Table S3) showed that the logistic regression and KNN analyses for SIB and property destruction made fewer correct classifications of *high severity behaviour*, with most individuals incorrectly classified as showing *no behaviou*r. The random forest classifier models showed slightly better performance and appeared more likely to make a Type I error of classifying participants showing *no behaviour* as at risk of showing *low* or *high severity behaviour*. The confusion matrixes showed models for aggression and any BtC showed a greater number of true positives and, importantly, fewer extreme false positives and false negatives than models for self‐injury and destruction of property.

Recall for presence of behaviour showed that the models could identify which individuals would show some level of behaviour (i.e., either *low* or *high severity behaviour*) ranging from 71.96% to 81.08%. Exceptions to this were the specific KNN and multinominal logistic regression models for self‐injurious behaviour and the KNN, multinominal logistic regression models and GMMs for property destruction, which showed comparatively poor performance. Recall for identifying the severity of behaviour ranged from 19.35% to 69.28%, which was lower and more variable than recall of presence of behaviour. This suggests that the models were able to identify true positive cases of individuals showing behaviour but were less sensitive in classifying the type of behaviour severity. These types of errors are reflected in the confusion matrixes. Across behaviour types, the random forest classifiers generally outperformed the multinominal logistic regression and KNN analyses for key metrics and in the number and severity of mistakes of classification being made.

Overall, examination of the internal validation analyses indicated that the random forest classifier model for any BtC was the best performing model. Based on the performance metrics for this model, 74.49% of individuals with *no behaviour*, 54.27% with *low severity behaviour* and 79.49% with *high severity behaviour* were correctly classified. Approximately 83.47% of people showing presence of any severity of behaviour would be identified as needing an intervention of which an estimated 94.87% of those at risk of the highest severity of behaviour would be included. Approximately 16.53% of people requiring an intervention would be ‘missed’ by the model, the majority of which show *low severity behaviour* (90.00%). Of individuals showing *no behaviour*, 25.68% would be incorrectly identified as showing risk for *low severity* or *high severity behaviour*.

### 3.2 External Validation

To explore how well the models could be used to predict risk of future levels of BtC, the models were applied to the longitudinal dataset T1 predictor variables, and accuracy was assessed against T1 and T2 outcomes. Performance metrics for all models at both time points are summarised in Tables S4–S6.

The AUCs for all models ranged from 0.620 to 0.730 at T1 outcome and 0.622 to 0.710 at T2 outcome, both of which were lower than the AUCs from internal validation and indicated fair predictability. Visual inspection of balanced accuracy, recall and precision metrics and confusion matrixes indicated no clear differences in the performances of all models to predict outcomes across the two timepoints. A decrease in the predictive performance of the models was observed compared to the internal validation analysis. Balanced accuracy decreased by up to 18.14% (random forest classifier for property destruction at T2), recall by 33.55% (KNN for property destruction at T2), precision by 37.40% (KNN for self‐injurious behaviour at T2) and AUC by 0.150 (random forest classifier for aggression at T2). However, these were the largest decreases in performance, and many models maintained a good level of predictability, such as the GMM for any BtC. This model showed a decrease of 2.65% for balanced accuracy, 7.18% for recall, 0.021 for AUC and even an increase of 2.33% for precision. Overall, analysis suggests that the models generalised well to the novel longitudinal external validation dataset, with some variability.

To explore the models' capacity to predict change in BtC over time, the persistence, incidence and remission of behaviour was calculated alongside percentages of correct prediction of these (see Tables S7 and S8). The number of incidences and remissions of behaviour in the one‐year period were low, meaning that the interpretation of these data is challenging. Similar to other performance metrics, these statistics showed that there was a high degree of variability in the models' ability to predict transitions in classifications over time. However, some models showed good performance, most notably the GMMs for self‐injurious behaviour, which identified 87.50% incidence of *high severity behaviour* from *low severity behaviour*, 50.00% of persistence of *no behaviour* and 100.00% persistence of *high severity behaviour*, although classification of persistence of *low severity behaviour* was comparatively poor (2.94%). The descriptive statistics of this analysis further demonstrate that the greatest classification error all models made was by categorising the *low severity behaviour* group incorrectly. This is further demonstrated in the confusion matrixes of the T1 and T2 outcome data (see Tables S6 and S7) and in the internal validation of the internal validation dataset (Table S3).

Similar to the findings from the internal validation, the random forest classifier was one of the better performing models across behaviour types. Though the performance of the GMMs improved with the GMM for any BtC being the best predictive model overall. Based on the performance metrics of the T2 analysis for any BtC, the GMM was able to correctly categorise 44.44% of *no behaviour*, 22.95% of *low severity behaviour* and 88.10% of *high severity behaviour* prevalence. Critically, using this model 83.50% of people showing any severity of behaviour would be identified as needing an intervention that includes 95.24% of those at risk of the highest severity of behaviour. The model indicates that 16.50% of people requiring an intervention would be ‘missed’ by the model, the majority of which show *low severity behaviour* (88.24%). Over time, the model was able to predict 60.00% persistence of *no behaviour*, 83.51% persistence of presence of behaviour and, most importantly, 83.33% incidence of behaviour. Of individuals showing *no behaviour*, 55.56% would be identified as showing risk for behaviour, broadly corresponding to a ‘misallocation of resources’ group.

### 3.3 Predictor Importance

Finally, to determine the contribution of individual variables in the development of predictive models of risk, coefficients of each variable and whether this significantly contributed to the model were calculated for multinomial logistic regressions (see Table S9) and Gini impurity indexes for the random forest classifier analyses (see Figure S1). Coefficients from the multinomial logistic regressions varied across behaviours with chronological age, impulsivity and overactivity contributing to all models. Differences in the variables contributing to predictions of *low severity behaviour* and *high severity behaviour* were observed across all models. This indicates that the *low* and *high severity behaviour* groups had distinct profiles of person characteristics that informed models of risk. Analyses of predictor importance for the random forest classifiers revealed that, similar to the multinomial logistic regressions, impulsivity, overactivity and chronological age emerged as the strongest predictors for all types of behaviour. Health problems and obsessive behaviour were identified as strongly predictive of self‐injurious behaviour, indicating some specificity of predictors for specific behaviours. The consistent predictive value of age in these models supports the need for service delivery to be targeted towards younger children to obtain more effective intervention outcomes.

## 4 Discussion

BtC are persistent and associated with poor outcomes for children with DD. Stratification of clinical services to deliver preventative interventions to children at highest risk of developing BtC is warranted, but progress towards this goal has been impeded by the absence of clinical tools and statistically defined models of risk. In this study, novel prediction models of risk for BtC were developed using machine learning approaches. The performance of these models was assessed in large datasets via internal validation and external validation on a 1‐year longitudinal community sample of individuals. Performance metrics indicated that all models had fair to good predictive ability, with some exceptions. Balanced accuracy, recall, precision and AUC all decreased when models were assessed on the external dataset; however, these still demonstrated that the models were able to make good classifications of risk for both current and future BtC outcomes. These findings represent an essential first step towards evidence‐based prevention and early intervention services for BtC.

This is the first study to utilise machine learning approaches to model risk of BtC in children with DD. Comparisons to the broader literature suggest the models performed similarly to those developed for anxiety, depression and psychosis by studies that are comparable in regards to the size of the dataset and number of outcomes of interest (Leighton et al. 2019; Priya et al. 2020). This represents an encouraging first step to modelling these complex behavioural characteristics in this population. Beyond this, there is limited guidance on what constitutes an appropriate cut‐off for performance metrics of a screening assessment of risk, particularly for multiple categorical outcomes. This is because the clinical implications of the predictions are important to consider, and so examination of confusion matrixes and attention to where ‘mistakes’ occur is advised when evaluating models (Watson and Petrie 2010). For making classifications of risk for BtC, identifying the presence of behaviour is arguably of greater importance than classifying the severity of behaviour. Pragmatically, it is necessary to ensure that children at risk for BtC are in contact with services before establishing what level of service input is required. Examination of the recall–presence statistic across the models (i.e., ability to identify presence of BoC irrespective of severity) suggests that the models have excellent predictive ability to identify the individuals for whom contact with services would be beneficial. For example, utilising the GMM for any BtC model in clinical practice would result in approximately 84% of individuals who require an intervention being identified. The strong predictive ability in the external validation analyses is encouraging as it highlights that, not only did the models generalise well to a novel dataset with significantly different background characteristics, but these models were also able to predict behaviour outcomes 1 year after screening. However, the cost of prioritising recall is that a greater number of individuals with ‘no risk’ were also likely to be identified as showing risk of BtC. There are negative consequences to incorrectly labelling someone as risk of *high severity behaviour*, which may include burden for the child and parent in participating in unnecessary interventions and potential stigmatisation. Nonetheless, the consequences of missing someone at risk for BtC may be more significant given its chronicity and significant negative impact on the child and the systems around them (Adams et al. 2016; Allen et al. 2009; Knapp et al. 2005; Laverty et al. 2020; McMahon et al. 2020; Beadle‐Brown et al. 2009; Richards et al. 2016). Therefore, future studies should further evaluate these prediction models, and any implementation in clinical practice should be cautious, with statistical models combining with clinical judgement as a tool to aid evidence‐based practice (Cutillo et al. 2020).

A key strength of the models presented here is the high rates of correct classifications of the *high severity behaviour* group, which represents the group with the greatest clinical need. Research has demonstrated that it is children with *high severity behaviour* who are most likely to require behaviour support from education and healthcare services and are more likely to be in contact with a social worker (Laverty et al. 2020; Ruddick et al. 2015). Although the models captured risk of *high severity behaviour* well, classification accuracy for the *low severity behaviour* group was lower. This may be because this group encompasses a broad range of behaviour severity and/or a variety of underlying mechanisms to behaviour (and consequently varying predictor importance), which the models were unable to account for effectively. Alternatively, it may be that the *low severity behaviour* group represents individuals that do not show a static presentation but rather one that changes dynamically over time. In typical development, some children are likely to show BtC that remits before age 5 years (Romanczyk et al. 1982). Even in the DD literature, not all BtC is persistent and may remit over time or after effective intervention (Laverty et al. 2020). Thus, the low severity group may encompass more variable or short‐term presentations of BtC, which can remit, persist or worsen to an incidence of *high severity behaviour*. Due to the heterogeneity of BtC and associated risk markers, modelling the complex pathways of behaviour is challenging in small clinical samples. Thus, large sample longitudinal studies are required to build more sophisticated prediction models.

Predictor importance was also explored for the logistic regression and random forest classifiers. This was to understand which factors fed into the construction of the models and to aid interpretation of model predictions to promote clinician confidence (Cutillo et al. 2020). Due to the nature of how KNN and GMM approaches operate, predictor importance could not be generated for these analyses. For the random forest classifiers and multinominal logistic regressions, a number of key predictors were identified as significant across the different models with variables of impulsivity, overactivity and chronological age consistently identified as important across all behaviour types and machine learning approaches. This is consistent with previous literature and, in the case of impulsivity, is particularly interesting as it lends support to theories of impaired behavioural inhibition, leading to BtC in people with autism and intellectual disability (McClintock et al. 2003; Oliver and Richards 2015; Schmitt et al. 2018). By comparison, predictor variables of adaptive behaviour, sex, health problems, autism and repetitive behaviour varied in their predictive significance based on the outcome behaviour of interest. For example, health problems were identified by the random forest classifiers as an important predictor for self‐injurious behaviour, but not for property destruction, aggressive behaviour or any BoC composite. This is supported by previous research that reported that the presence of painful health conditions has a stronger association with self‐injurious behaviour than other BtC types (Symons and Danov 2005; Walsh et al. 2011). This highlights the need for specificity in behaviour outcomes when building prediction models of risk, as the underlying mechanisms for specific behaviours may vary and the inclusion of unique predictor variables for specific behaviours may enhance the subsequent accuracy of models.

When evaluating the models, it is important to note that predictive ability may have been limited by the number and type of risk markers selected. Predictor variables focussed predominantly on child characteristics rather than wider caregiver characteristics or environmental factors. This may have limited the accuracy of modelling more specific behaviours where more nuanced pathways to behaviour and environmental interactions exist. Future algorithms may seek to capture these variables, but this should be balanced with a need for a screening measure that is short and easy to complete. Similarly, the analytical approaches in the current study were limited by the size of the dataset and computational costs. Future research should seek to replicate and extend these findings in larger datasets by evaluating different combinations of these features and other features not measured in the present study to examine the impact on the classification metrics. Such analyses could usefully expose all statistical models to the same set of permutations, employing Bayesian model evidence to evaluate model fit.

There are several limitations of this study that should also be considered. Firstly, although the sample sizes were sufficient to run the analyses, the heterogeneous nature of the behaviours under investigation alongside the small number of individuals falling into some categories may have weakened statistical power. Similarly, analyses examining the ability of the models to predict incidence of behaviour were significantly limited by sample size. This is a common limitation of identification of risk markers for BtC in the broader literature, as previous research also shows low incidence rates (Davies and Oliver 2016; Laverty et al. 2020). Thus, in order to robustly identify risk of BtC, there is a need for future studies to recruit larger samples and utilise longitudinal designs to better capture incidence of BtC and model more nuanced pathways to specific behaviour types.

In summary, the findings presented here represent an essential step towards developing prediction models of risk for BtC. The results indicate that such models may be effective for translation to clinical practice to inform clinicians as to which individuals may require early preventative intervention. Further refinement and validation of the models are required, as well as consideration of the applicability to clinical practice. Nonetheless, such an approach may offer a useful tool to services to develop a more proactive, preventative healthcare system for individuals with DD.

## Funding

This work was funded by the British Academy of Childhood Disability‐Castang Foundation for funding this project. This work has also received funding from the European Research Council (ERC) under the Horizon 2020 Framework Programme (CartographY GA. 804752) and Cerebra.

## Ethics Statement

Full ethical approval for the study was received from North East ‐ Tyne & Wear South Research Ethics Committee (reference: RG_17‐182).

## Conflicts of Interest

The authors declare no conflicts of interest.

## Supporting information


**Table S1:** Predictor variables in SAD‐SQ calculated in accordance with Davies and Oliver (2016): item descriptions, measurement and values.
**Table 2.** Performance metrics means and confidence intervals for the internal validation of prediction models for all behaviour types.
**Table S3:** Confusion matrixes from the internal validation of prediction models for all behaviour types. Green cells indicate a correct classification (true positive or true negative), amber cells indicate a misclassification (false positive or false negative one cell from target), and red indicates an extreme misclassification (false positive or false negative two cells from target).
**Table S4:** Performance metrics' means and confidence intervals for the external validation of prediction models on Time 1 and Time 2 data for each model for all behaviour types.
**Table S5:** Confusion matrixes from the external validation of prediction models on Time 1 data for all behaviour types. Green cells indicate a correct classification (true positive or true negative), amber cells indicate a misclassification (false positive or false negative one cell from target), and red indicates an extreme misclassification (false positive or false negative two cells from target).
**Table S6:** Confusion matrixes from the external validation of prediction models on Time 2 data for all behaviour types. Green cells indicate a correct classification (true positive or true negative), amber cells indicate a misclassification (false positive or false negative one cell from target), and red indicates an extreme misclassification (false positive or false negative two cells from target).
**Table S7:** Confusion matrixes from the external validation of prediction models showing number and percentage of correct classifications of incidence, persistence and remission of behaviour from Time 1 to Time 2 for all behaviour types. Green cells indicate a correct classification (true positive or true negative), amber cells indicate a misclassification (false positive or false negative one cell from target), and red indicates an extreme misclassification (false positive or false negative two cells from target).
**Table S8:** External validation of prediction models showing percentage of correct classifications of incidence, persistence and remission of behaviour from Time 1 to Time 2 for presence of behaviour (no behaviour versus low and high behaviour composite group) and severity of behaviour (low behaviour versus high behaviour).
**Table S9:** Coefficients and significance of predictor variables for multinominal logistic regression models of self‐injurious behaviour, aggression, destruction of property and any BtC.
**Figure S1:** Relative importance (mean decrease in Gini impurity) of predictor variables and 95% confidence intervals for random forest models of self‐injurious behaviour, aggression, destruction of property and any BtC.

## Data Availability

The data that support the findings of this study are available on request from the corresponding author.

## Endnotes

^1^
Unpublished dataset. Recruitment was conducted through Child Development Centres using a similar approach to Davies and Oliver (2016).
