# Supplementary material for: The Development and Validation of Models of Risk for Behaviours That Challenge in Children With Developmental Disabilities: A Novel Machine Learning Approach
Source: J Intellect Disabil Res. 2026 Apr 21;70(7):700–8. doi: 10.1111/jir.70105 (PMC13238332; doi:10.1111/jir.70105)
Supplement: Supplementary file 1 — Table S1: Predictor variables in SAD‐SQ calculated in accordance with Davies and Oliver (2016): item descriptions, measurement and values. Table 2. Performance metrics means and confidence intervals for the internal validation of prediction models for all behaviour types. Table S3: Confusion matrixes from the internal validation of prediction models for all behaviour types. Green cells indicate a correct classification (true positive or true negative), amber cells indicate a misclassification (false positive or false negative one cell from target), and red indicates an extreme misclassification (false positive or false negative two cells from target). Table S4: Performance metrics' means and confidence intervals for the external validation of prediction models on Time 1 and Time 2 data for each model for all behaviour types. Table S5: Confusion matrixes from the external validation of prediction models on Time 1 data for all behaviour types. Green cells indicate a correct classification (true positive or true negative), amber cells indicate a misclassification (false positive or false negative one cell from target), and red indicates an extreme misclassification (false positive or false negative two cells from target). Table S6: Confusion matrixes from the external validation of prediction models on Time 2 data for all behaviour types. Green cells indicate a correct classification (true positive or true negative), amber cells indicate a misclassification (false positive or false negative one cell from target), and red indicates an extreme misclassification (false positive or false negative two cells from target). Table S7: Confusion matrixes from the external validation of prediction models showing number and percentage of correct classifications of incidence, persistence and remission of behaviour from Time 1 to Time 2 for all behaviour types. Green cells indicate a correct classification (true positive or true negative), amber cells indicate a misclassification (f [file JIR-70-700-s001.docx]

**Supplementary Table 1.** Predictor variables in SAD-SQ calculated in accordance with Davies & Oliver (2016): item descriptions, measurement and values

| **Variable** | **Item description** | **Data type** | **Possible score range** |
| --- | --- | --- | --- |
| Adaptive behaviour | Parents and caregivers answered twenty items from the Denver Developmental Screening Test II (DDST II; [Frankenburg, Dodds, Archer, Shapiro, & Bresnick, 1992](https://www.sciencedirect.com/science/article/pii/S0891422215300251?casa_token=exMSv2d_er8AAAAA:Usf7QYkjBql39I2WmneKCdYtoccOz8r_EeA5tXLV2-yxMY_LutuMliNusvyKjcIapEuHnNuuXQ#bib0081)) (children under six) or three items from the Wessex Behaviour Scale ([Kushlick, Blunden, & Cox, 1973](https://www.sciencedirect.com/science/article/pii/S0891422215300251?casa_token=exMSv2d_er8AAAAA:Usf7QYkjBql39I2WmneKCdYtoccOz8r_EeA5tXLV2-yxMY_LutuMliNusvyKjcIapEuHnNuuXQ#bib0145)) (children aged six years and over). A median split was performed separately on the data from children under six and children six and over to classify all children into high and low adaptive behaviour groups (median split for low ability on Denver < 29, median split for low ability on Wessex < 8) | Binary (high/low adaptive behaviour) | 0 - 1 |
| Autism | Parents and caregivers were asked whether any health professional has said that your child is autistic, has an autism spectrum disorder, autistic like traits and/or features of autism. | Binary (yes/no) | 0 – 1 |
| Number of health problems | Common health problems were listed (eye, ear, dental, digestive, skin, respiratory, epilepsy, ‘other’) and the number of endorsed items was used as the measure of health of health problems. | Continuous | 0-6 |
| Repetitive behaviour | Two items from the behaviour and emotional difficulties section of the Self-Help and [Behaviour Rating Scale](https://www.sciencedirect.com/topics/psychology/behavior-rating-scale) ([Petty, 2006](https://www.sciencedirect.com/science/article/pii/S0891422215300251?casa_token=exMSv2d_er8AAAAA:Usf7QYkjBql39I2WmneKCdYtoccOz8r_EeA5tXLV2-yxMY_LutuMliNusvyKjcIapEuHnNuuXQ#bib0220)) pertaining to repetitive movements were summed to assess repetitive and restricted behaviour. | Ordinal | 0-8 |
| Obsessive behaviour | Two items from the behaviour and emotional difficulties section of the Self-Help and Behaviour Rating Scale (Petty, 2006) obsessions and rituals were used to assess obsessive behaviour. | Ordinal | 0-8 |
| Impulsivity | Two items from the Activity Questionnaire ([Burbidge et al., 2010](https://www.sciencedirect.com/science/article/pii/S0891422215300251?casa_token=exMSv2d_er8AAAAA:Usf7QYkjBql39I2WmneKCdYtoccOz8r_EeA5tXLV2-yxMY_LutuMliNusvyKjcIapEuHnNuuXQ#bib0035)) pertaining to difficulties waiting and wanting things immediately were summed in order to assess impulsive behaviour | Ordinal | 0-8 |
| Overactivity | Two items from the Activity Questionnaire ([Burbidge et al., 2010](https://www.sciencedirect.com/science/article/pii/S0891422215300251?casa_token=exMSv2d_er8AAAAA:Usf7QYkjBql39I2WmneKCdYtoccOz8r_EeA5tXLV2-yxMY_LutuMliNusvyKjcIapEuHnNuuXQ#bib0035)) pertaining to acting as if driven by a motor and finding it difficult to hold still were summed to assess overactive and behaviour | Ordinal | 0-8 |

| **Supplementary Table 2.** Performance metrics means and confidence intervals for the internal validation of prediction models for all behaviour types. | | | | | | | | | | | | | | | | | | | | | | | | | | | |  |
| --- | --- | --- | --- | --- | --- | --- | --- | --- | --- | --- | --- | --- | --- | --- | --- | --- | --- | --- | --- | --- | --- | --- | --- | --- | --- | --- | --- | --- |
|  | |  | | | | **% Balanced Accuracy**  **[95% CI]** | | | | | **% Recall**  **[95% CI]** | | | **% Precision**  **[95% CI]** | | | **AUC**  **[95% CI]** | | | **% Recall-presence [95% CI]** | | | | **% Recall-level**  **[95% CI]** | | | |  |
| **Self-injurious behaviour** | | KNN | | | | 37.25  [34.13, 41.54] | | | | | 67.85  [64.06, 71.24] | | | 59.43  [52.25, 67.16] | | | .700  [.665, .732] | | | 20.01  [8.77, 34.01] | | | | NA^a^  [0.00, 100.00] | | | |  |
|  |  | Logistic regression | | | | 37.59  [33.69, 41.80] | | | | | 66.11  [62.64, 69.92] | | | 56.24  [46.88-65.35] | | | .696  [.663-.731] | | | 16.06  [5.04, 30.40] | | | | 51.97  [0.00, 100.00] | | | |  |
|  |  | GMM | | | | 52.03  [47.19, 57.14] | | | | | 57.51  [53.40, 61.66] | | | 56.24  [46.88-65.35] | | | .696  [.663-.731] | | | 75.44  [67.60, 82.71] | | | | 36.11  [19.14, 54.07] | | | |  |
|  |  | Random Forest | | | | 52.73  [46.82, 58.59] | | | | | 60.57  [55.32, 65.45] | | | 57.61  [53.14, 62.24] | | | .733  [.697, .766] | | | 71.96  [62.26, 81.00] | | | | 58.92  [38.22, 77.43] | | | |  |
| **Aggression** | | KNN | | | | 51.82  [46.71, 57.27] | | | | | 59.55  [55.55, 63.32] | | | 59.28  [54.78, 64.14] | | | .757  [.727, .786] | | | 73.61  [63.92, 82.68] | | | | 27.77  [9.43, 50.00] | | | |  |
|  | | Logistic regression | | | | 52.01  [47.45, 57.10] | | | | | 59.30  [55.32, 63.28] | | | 58.90  [54.30, 63.49] | | | .752  [.723, .779] | | | 72.83  [64.09, 80.70] | | | | 28.70  [11.32, 48.58] | | | |  |
|  | | GMM | | | | 52.03  [47.19, 57.14] | | | | | 57.51  [53.40, 61.66] | | | 57.61  [53.14, 62.24] | | | .733  [.697, .766] | | | 75.44  [67.60, 82.71] | | | | 36.12  [19.14, 54.07] | | | |  |
|  |  | Random Forest | | | | 59.72  [54.98, 64.12] | | | | | 58.51  [54.08, 62.60] | | | 61.19  [56.70, 65.47] | | | .781  [.750, .808] | | | 80.25  [73.20, 87.31] | | | | 66.98  [51.85, 80.44] | | | |  |
| **Destruction of property** | | KNN | | | | 47.25  [42.75, 52.37] | | | | | 65.78  [61.92, 69.45] | | | 62.87  [56.76, 68.48] | | | .769  [.739, .798] | | | 52.63  [39.21, 65.19] | | | | 19.35  [0.00, 47.38] | | | |  |
|  |  | Logistic regression | | | | 47.70  [42.42, 53.48] | | | | | 64.08  [60.17, 68.08] | | | 61.36  [55.90, 66.53] | | | .762  [.735, .787] | | | 52.68  [39.62, 64.18] | | | | 30.69  [7.14, 58.83] | | | |  |
|  | | GMM | | | | 47.78  [42.56, 52.60] | | | | | 61.44  [57.10, 65.96] | | | 60.24  [54.64, 65.51] | | | .732  [.693, .765] | | | 58.44  [46.93, 69.07] | | | | 37.75  [18.18, 59.26] | | | |  |
|  |  | Random Forest | | | | 58.50  [53.42, 63.45] | | | | | 62.65  [58.47, 67.11] | | | 67.53  [63.24, 71.49] | | | .760  [.757, .791] | | | 78.95  [70.63, 86.76] | | | | 62.74  [45.65, 77.78] | | | |  |
| **Any BtC** | | KNN | | | | 55.58  [50.66, 60.29] | | | | | 58.03  [53.81, 61.91] | | | 58.41  [53.98, 62.68] | | | .756  [.733, .787] | | | 79.38  [70.41, 87.24] | | | | 43.12  [25.29, 61.77] | | | |  |
|  |  | Logistic regression | | | | 56.36  [51. 74, 61.30] | | | | | 58.54  [54.40, 62.70] | | | 58.84  [54.49, 63.06] | | | .760  [.731, .787] | | | 79.26  [71.60, 85.95] | | | | 45.81  [28.37, 62,70] | | | |  |
|  | | GMM | | | | 54.48  [49.51, 58.91] | | | | | 55.94  [51.63, 60.11] | | | 56.26  [51.64, 60.64] | | | .731  [.700, .763] | | | 80.24  [73.28, 86.08] | | | | 47.04  [32.83, 62.50] | | | |  |
|  |  | Random Forest | | | | 59.93  [55.59, 63.83] | | | | | 58.20  [54.21, 62.04] | | | 59.12  [54.94, 63.08] | | | .782  [.757, .808] | | | 81.08  [73.86, 87.50] | | | | 69.28  [56.79, 80.95] | | | |  |
| *Note:* AUC = Area Under the Curve; Recall-presence = recall metric for presence of behaviour (i.e., no behaviour versus low and high behaviour composite group); Recall-level = recall metric for severity of behaviour (i.e., low behaviour versus high behaviour).  ^a^ Not Applicable, mean could not be calculated due to no counts of prediction data within some iterations of bootstrapped data | | | | | | | | | | | | | | | | | | | | | | | | | | | |  |
| **Supplementary Table 3.** Confusion matrixes from the internal validation of prediction models for all behaviour types. Green cells indicate a correct classification (true positive or true negative), amber cells indicate a misclassification (false positive or false negative one cell from target), and red indicates an extreme misclassification (false positive or false negative two cells from target). | | | | | | | | | | | | | | | | | | | | | | | | | | | | |
|  | **K-Nearest Neighbours** | | | | | | | **Logistic Regression** | | | | | | | **GMM** | | | | | | | **Random Forest** | | | | | | |
| **Self-injurious behaviour** |  | |  |  |  | |  |  |  |  | |  |  | |  |  | |  |  | |  |  |  | |  |  |  | |
|  |  | |  | Predicted | | | |  |  | Predicted | | | | |  |  | | Predicted | | | |  |  | | Predicted | | | |
|  |  | |  | No | Low | | High |  |  | No | | Low | High | |  |  | | No | Low | | High |  |  | | No | Low | High | |
|  | Actual | | No | 524 | 17 | | 1 | Actual | No | 522 | | 11 | 9 | | Actual | No | | 495 | 21 | | 26 | Actual | No | | 384 | 83 | 75 | |
|  |  |  | Low | 132 | 24 | | 2 |  | Low | 144 | | 7 | 7 | |  | Low | | 107 | 38 | | 13 |  | Low | | 36 | 93 | 29 | |
|  |  |  | High | 57 | 12 | | 5 |  | High | 57 | | 5 | 12 | |  | High | | 36 | 9 | | 29 |  | High | | 4 | 9 | 61 | |
|  |  | |  |  |  | |  |  |  |  | |  |  | |  |  | |  |  | |  |  |  | |  |  |  | |
| **Aggression** |  | |  | Predicted | | | |  |  | Predicted | | | | |  |  | | Predicted | | | |  |  | | Predicted | | | |
|  |  | |  | No | Low | | High |  |  | No | | Low | High | |  |  | | No | Low | | High |  |  | | No | Low | High | |
|  | Actual | | No | 246 | 85 | | 9 | Actual | No | 256 | | 76 | 8 | | Actual | No | | 244 | 82 | | 14 | Actual | No | | 237 | 73 | 30 | |
|  |  |  | Low | 84 | 218 | | 18 |  | Low | 106 | | 201 | 13 | |  | Low | | 86 | 199 | | 35 |  | Low | | 61 | 180 | 79 | |
|  |  |  | High | 10 | 62 | | 34 |  | High | 7 | | 70 | 29 | |  | High | | 7 | 47 | | 52 |  | High | | 3 | 17 | 86 | |
|  |  | |  |  |  | |  |  |  |  | |  |  | |  |  | |  |  | |  |  |  | |  |  |  | |
| **Destruction of Property** |  | |  | Predicted | | | |  |  | Predicted | | | | |  |  | | Predicted | | | |  |  | | Predicted | | | |
|  |  | |  | No | Low | | High |  |  | No | | Low | High | |  |  | | No | Low | | High |  |  | | No | Low | High | |
|  | Actual | | No | 423 | 49 | | 3 | Actual | No | 414 | | 54 | 7 | | Actual | No | | 401 | 55 | | 19 | Actual | No | | 351 | 82 | 42 | |
|  |  |  | Low | 100 | 104 | | 7 |  | Low | 118 | | 82 | 11 | |  | Low | | 93 | 93 | | 25 |  | Low | | 40 | 128 | 43 | |
|  |  |  | High | 35 | 38 | | 12 |  | High | 20 | | 47 | 18 | |  | High | | 14 | 30 | | 41 |  | High | | 5 | 14 | 66 | |
|  |  | |  |  |  | |  |  |  |  | |  |  | |  |  | |  |  | |  |  |  | |  |  |  | |
| **Any BtC** |  | |  | Predicted | | | |  |  | Predicted | | | | |  |  | | Predicted | | | |  |  | | Predicted | | | |
|  |  | |  | No | Low | | High |  |  | No | | Low | High | |  |  | | No | Low | | High |  |  | | No | Low | High | |
|  | Actual | | No | 215 | 69 | | 10 | Actual | No | 210 | | 75 | 9 | | Actual | No | | 209 | 69 | | 16 | Actual | No | | 219 | 56 | 19 | |
|  |  |  | Low | 75 | 220 | | 33 |  | Low | 81 | | 207 | 40 | |  | Low | | 72 | 203 | | 53 |  | Low | | 72 | 178 | 78 | |
|  |  |  | High | 9 | 76 | | 71 |  | High | 9 | | 78 | 69 | |  | High | | 3 | 62 | | 91 |  | High | | 8 | 24 | 124 | |
|  |  | |  |  |  | |  |  |  |  | |  |  | |  |  | |  |  | |  |  |  | |  |  |  | |

| **Supplementary Table 4.** Performance metrics’ means and confidence intervals for the external validation of prediction models on Time 1 and Time 2 data for each model for all behaviour types. | | | | | | | |
| --- | --- | --- | --- | --- | --- | --- | --- |
|  |  | **% Balanced Accuracy** | **% Recall** | **% Precision** | **AUC** | **% Recall-presence** | **% Recall-level** |
| Time 1 data | | | | | | | |
| **Self-injurious behaviour** | KNN | 39.09 | 45.45 | 26.41 | .665 | 11.76 | 100.00 |
|  | Logistic regression | 32.13 | 42.15 | 32.71 | .629 | 5.88 | 10.53 |
|  | GMM | 47.40 | 35.54 | 42.53 | .648 | 82.35 | 94.74 |
|  | Random Forest | 48.43 | 41.32 | 39.71 | .620 | 62.26 | 8.33 |
| **Aggression** | KNN | 41.13 | 37.19 | 45.13 | .652 | 45.74 | 30.00 |
|  | Logistic regression | 47.41 | 46.28 | 49.34 | .680 | 64.89 | 30.77 |
|  | GMM | 52.69 | 47.93 | 52.52 | .721 | 88.30 | 87.88 |
|  | Random Forest | 48.73 | 45.45 | 50.52 | .667 | 61.70 | 56.00 |
| **Destruction of property** | KNN | 35.89 | 40.50 | 38.33 | .724 | 16.00 | 20.00 |
|  | Logistic regression | 49.83 | 52.07 | 52.47 | .718 | 84.78 | 61.54 |
|  | GMM | 44.81 | 42.15 | 47.87 | .700 | 88.00 | 54.17 |
|  | Random Forest | 40.36 | 42.98 | 39.70 | .687 | 44.00 | 38.46 |
| **Any BtC** | KNN | 49.83 | 42.15 | 53.54 | .668 | 59.62 | 48.48 |
|  | Logistic regression | 50.37 | 47.11 | 52.61 | .701 | 72.12 | 78.95 |
|  | GMM | 50.90 | 48.76 | 52.00 | .730 | 82.69 | 95.35 |
|  | Random Forest | 52.27 | 45.45 | 55.12 | .672 | 64.42 | 72.73 |
| Time 2 data | | | | | | | |
| **Self-injurious behaviour** | KNN | 38.93 | 40.50 | 22.03 | .663 | 10.81 | 100.00 |
|  | Logistic regression | 33.84 | 39.67 | 45.98 | .643 | 8.11 | 0.00 |
|  | GMM | 49.71 | 36.36 | 46.19 | .628 | 83.78 | 94.74 |
|  | Random Forest | 52.58 | 42.15 | 44.60 | .643 | 60.81 | 94.12 |
| **Aggression** | KNN | 40.15 | 36.36 | 44.17 | .622 | 45.26 | 29.41 |
|  | Logistic regression | 41.56 | 40.50 | 43.72 | .630 | 62.11 | 26.92 |
|  | GMM | 47.09 | 42.15 | 47.10 | .651 | 85.26 | 86.67 |
|  | Random Forest | 49.08 | 45.45 | 51.72 | .631 | 86.57 | 56.00 |
| **Destruction of property** | KNN | 32.42 | 32.23 | 30.81 | .639 | 11.11 | 33.33 |
|  | Logistic regression | 40.02 | 39.67 | 40.99 | .638 | 45.68 | 38.46 |
|  | GMM | 45.99 | 41.32 | 49.56 | .658 | 81.48 | 63.16 |
|  | Random Forest | 40.36 | 42.98 | 39.70 | .687 | 44.00 | 38.46 |
| **Any BtC** | KNN | 41.04 | 33.88 | 41.01 | .631 | 58.25 | 38.71 |
|  | Logistic regression | 50.28 | 45.45 | 48.86 | .709 | 72.82 | 76.32 |
|  | GMM | 51.83 | 48.76 | 58.59 | .710 | 83.50 | 92.50 |
|  | Random Forest | 48.43 | 41.32 | 48.29 | .698 | 64.08 | 66.67 |
| *Note:* AUC = Area Under the Curve; Recall-presence = recall metric for presence of behaviour (i.e., no behaviour versus low and high behaviour composite group); Recall-level = recall metric for severity of behaviour (i.e., low behaviour versus high behaviour). | | | | | | | |

| **Supplementary Table 5.** Confusion matrixes from the external validation of prediction models on Time 1 data for all behaviour types. Green cells indicate a correct classification (true positive or true negative), amber cells indicate a misclassification (false positive or false negative one cell from target), and red indicates an extreme misclassification (false positive or false negative two cells from target). | | | | | | | | | | | | | | | | | | | | |
| --- | --- | --- | --- | --- | --- | --- | --- | --- | --- | --- | --- | --- | --- | --- | --- | --- | --- | --- | --- | --- |
|  | **K-Nearest Neighbours** | | | | | **Logistic Regression** | | | | | **GMM** | | | | | **Random Forest** | | | | |
| **Self-injurious behaviour** |  |  |  |  |  |  |  |  |  |  |  |  |  |  |  |  |  |  |  |  |
|  |  |  | Predicted | | |  |  | Predicted | | |  |  | Predicted | | |  |  | Predicted | | |
|  |  |  | No | Low | High |  |  | No | Low | High |  |  | No | Low | High |  |  | No | Low | High |
|  | Actual | No | 51 | 0 | 2 | Actual | No | 50 | 0 | 3 | Actual | No | 23 | 5 | 25 | Actual | No | 33 | 4 | 16 |
|  |  | Low | 45 | 0 | 4 |  | Low | 47 | 1 | 1 |  | Low | 12 | 2 | 35 |  | Low | 25 | 2 | 22 |
|  |  | High | 15 | 0 | 4 |  | High | 1 | 17 | 2 |  | High | 0 | 1 | 18 |  | High | 3 | 1 | 15 |
|  |  |  |  |  |  |  |  |  |  |  |  |  |  |  |  |  |  |  |  |  |
| **Aggression** |  |  | Predicted | | |  |  | Predicted | | |  |  | Predicted | | |  |  | Predicted | | |
|  |  |  | No | Low | High |  |  | No | Low | High |  |  | No | Low | High |  |  | No | Low | High |
|  | Actual | No | 20 | 5 | 2 | Actual | No | 19 | 6 | 2 | Actual | No | 12 | 8 | 7 | Actual | No | 18 | 6 | 3 |
|  |  | Low | 37 | 19 | 4 |  | Low | 25 | 29 | 6 |  | Low | 10 | 17 | 33 |  | Low | 27 | 23 | 10 |
|  |  | High | 14 | 14 | 6 |  | High | 8 | 18 | 8 |  | High | 1 | 4 | 29 |  | High | 9 | 11 | 14 |
|  |  |  |  |  |  |  |  |  |  |  |  |  |  |  |  |  |  |  |  |  |
| **Destruction of Property** |  |  | Predicted | | |  |  | Predicted | | |  |  | Predicted | | |  |  | Predicted | | |
|  |  |  | No | Low | High |  |  | No | Low | High |  |  | No | Low | High |  |  | No | Low | High |
|  | Actual | No | 44 | 1 | 1 | Actual | No | 39 | 6 | 1 | Actual | No | 27 | 10 | 9 | Actual | No | 38 | 7 | 1 |
|  |  | Low | 44 | 4 | 3 |  | Low | 25 | 16 | 10 |  | Low | 9 | 11 | 31 |  | Low | 31 | 9 | 11 |
|  |  | High | 19 | 4 | 1 |  | High | 10 | 6 | 8 |  | High | 0 | 11 | 13 |  | High | 11 | 8 | 5 |
|  |  |  |  |  |  |  |  |  |  |  |  |  |  |  |  |  |  |  |  |  |
| **Any BtC** |  |  | Predicted | | |  |  | Predicted | | |  |  | Predicted | | |  |  | Predicted | | |
|  |  |  | No | Low | High |  |  | No | Low | High |  |  | No | Low | High |  |  | No | Low | High |
|  | Actual | No | 13 | 3 | 1 | Actual | No | 9 | 6 | 2 | Actual | No | 7 | 5 | 4 | Actual | No | 12 | 3 | 2 |
|  |  | Low | 31 | 22 | 7 |  | Low | 23 | 18 | 19 |  | Low | 17 | 11 | 32 |  | Low | 26 | 19 | 15 |
|  |  | High | 11 | 17 | 16 |  | High | 6 | 8 | 30 |  | High | 1 | 2 | 41 |  | High | 11 | 9 | 24 |
|  |  |  |  |  |  |  |  |  |  |  |  |  |  |  |  |  |  |  |  |  |

| **Supplementary Table 6.** Confusion matrixes from the external validation of prediction models on Time 2 data for all behaviour types. Green cells indicate a correct classification (true positive or true negative), amber cells indicate a misclassification (false positive or false negative one cell from target), and red indicates an extreme misclassification (false positive or false negative two cells from target). | | | | | | | | | | | | | | | | | | | | |
| --- | --- | --- | --- | --- | --- | --- | --- | --- | --- | --- | --- | --- | --- | --- | --- | --- | --- | --- | --- | --- |
|  | **K-Nearest Neighbours** | | | | | **Logistic Regression** | | | | | **GMM** | | | | | **Random Forest** | | | | |
| **Self-injurious behaviour** |  |  |  |  |  |  |  |  |  |  |  |  |  |  |  |  |  |  |  |  |
|  |  |  | Predicted | | |  |  | Predicted | | |  |  | Predicted | | |  |  | Predicted | | |
|  |  |  | No | Low | High |  |  | No | Low | High |  |  | No | Low | High |  |  | No | Low | High |
|  | Actual | No | 45 | 0 | 2 | Actual | No | 46 | 0 | 1 | Actual | No | 4 | 20 | 12 | Actual | No | 32 | 3 | 12 |
|  |  | Low | 51 | 0 | 4 |  | Low | 50 | 2 | 3 |  | Low | 3 | 40 | 0 |  | Low | 27 | 3 | 25 |
|  |  | High | 15 | 0 | 4 |  | High | 18 | 1 | 0 |  | High | 0 | 1 | 18 |  | High | 2 | 1 | 16 |
|  |  |  |  |  |  |  |  |  |  |  |  |  |  |  |  |  |  |  |  |  |
| **Aggression** |  |  | Predicted | | |  |  | Predicted | | |  |  | Predicted | | |  |  | Predicted | | |
|  |  |  | No | Low | High |  |  | No | Low | High |  |  | No | Low | High |  |  | No | Low | High |
|  | Actual | No | 19 | 6 | 1 | Actual | No | 16 | 8 | 2 | Actual | No | 9 | 9 | 8 | Actual | No | 17 | 7 | 2 |
|  |  | Low | 37 | 20 | 6 |  | Low | 30 | 26 | 7 |  | Low | 12 | 16 | 35 |  | Low | 28 | 24 | 11 |
|  |  | High | 15 | 12 | 5 |  | High | 6 | 19 | 7 |  | High | 2 | 4 | 26 |  | High | 9 | 9 | 14 |
|  |  |  |  |  |  |  |  |  |  |  |  |  |  |  |  |  |  |  |  |  |
| **Destruction of Property** |  |  | Predicted | | |  |  | Predicted | | |  |  | Predicted | | |  |  | Predicted | | |
|  |  |  | No | Low | High |  |  | No | Low | High |  |  | No | Low | High |  |  | No | Low | High |
|  | Actual | No | 35 | 4 | 1 | Actual | No | 30 | 7 | 3 | Actual | No | 21 | 8 | 11 | Actual | No | 28 | 10 | 2 |
|  |  | Low | 54 | 3 | 3 |  | Low | 36 | 13 | 11 |  | Low | 13 | 17 | 30 |  | Low | 41 | 8 | 11 |
|  |  | High | 18 | 2 | 1 |  | High | 8 | 8 | 5 |  | High | 2 | 7 | 12 |  | High | 11 | 6 | 4 |
|  |  |  |  |  |  |  |  |  |  |  |  |  |  |  |  |  |  |  |  |  |
| **Any BtC** |  |  | Predicted | | |  |  | Predicted | | |  |  | Predicted | | |  |  | Predicted | | |
|  |  |  | No | Low | High |  |  | No | Low | High |  |  | No | Low | High |  |  | No | Low | High |
|  | Actual | No | 12 | 6 | 0 | Actual | No | 10 | 7 | 1 | Actual | No | 8 | 2 | 8 | Actual | No | 12 | 4 | 2 |
|  |  | Low | 32 | 17 | 12 |  | Low | 24 | 16 | 21 |  | Low | 15 | 14 | 32 |  | Low | 28 | 16 | 17 |
|  |  | High | 11 | 19 | 12 |  | High | 4 | 9 | 29 |  | High | 2 | 3 | 37 |  | High | 9 | 11 | 22 |
|  |  |  |  |  |  |  |  |  |  |  |  |  |  |  |  |  |  |  |  |  |

| **Supplementary Table 7.**  Confusion matrixes from the external validation of prediction models showing number and percentage of correct classifications of incidence, persistence and remission of behaviour from Time 1 to Time 2 for all behaviour types. Green cells indicate a correct classification (true positive or true negative), amber cells indicate a misclassification (false positive or false negative one cell from target), and red indicates an extreme misclassification ( false positive or false negative two cells from target). | | | | | | | | | | | | | | | | | | | | | | |
| --- | --- | --- | --- | --- | --- | --- | --- | --- | --- | --- | --- | --- | --- | --- | --- | --- | --- | --- | --- | --- | --- | --- |
|  |  |  |  | **KNN risk predictions** | | | |  | **Multinomial logistic regression risk predictions** | | | |  | **Gaussian Mixture Models risk predictions** | | | |  | **Random forest classifiers**  **risk predictions** | | | |
|  |  |  |  |  |  |  |  |  |  |  |  |  |  |  |  |  |  |  |  |  |  |  |
|  | T1 behaviour | T2 outcome | Total *n* | No | Low | High | % correct |  | No | Low | High | % correct |  | No | Low | High | % correct |  | No | Low | High | % correct |
| **Self injurious behaviour** |  |  |  |  |  |  |  |  |  |  |  |  |  |  |  |  |  |  |  |  |  |  |
|  | **No behaviour** | Persistence | 40 | 39 | 0 | 1 | 97.50 |  | 39 | 0 | 1 | 97.50 |  | 20 | 4 | 16 | 50.00 |  | 27 | 3 | 10 | 67.50 |
|  |  | Incidence (low severity) | 13 | 12 | 0 | 1 | 0.00 |  | 11 | 0 | 2 | 0.00 |  | 3 | 1 | 9 | 7.69 |  | 6 | 1 | 6 | 7.69 |
|  |  | Incidence (high severity) | 0 | 0 | 0 | 0 | NA |  | 0 | 0 | 0 | NA |  | 0 | 0 | 0 | NA |  | 0 | 0 | 0 | NA |
|  |  |  |  |  |  |  |  |  |  |  |  |  |  |  |  |  |  |  |  |  |  |  |
|  | **Low severity** | Remission (no behaviour) | 7 | 6 | 0 | 1 | 85.71 |  | 7 | 0 | 0 | 100.00 |  | 3 | 0 | 4 | 42.86 |  | 5 | 0 | 2 | 71.43 |
|  |  | Persistence | 34 | 32 | 0 | 2 | 0.00 |  | 32 | 1 | 1 | 2.94 |  | 9 | 1 | 24 | 2.94 |  | 18 | 2 | 14 | 5.88 |
|  |  | Incidence (high severity) | 8 | 7 | 0 | 1 | 12.50 |  | 8 | 0 | 0 | 0.00 |  | 0 | 1 | 7 | 87.50 |  | 2 | 0 | 6 | 75.00 |
|  |  |  |  |  |  |  |  |  |  |  |  |  |  |  |  |  |  |  |  |  |  |  |
|  | **High severity** | Remission (no behaviour) | 0 | 0 | 0 | 0 | NA |  | 0 | 0 | 0 | NA |  | 0 | 0 | 0 | NA |  | 0 | 0 | 0 | NA |
|  |  | Remission (low severity) | 8 | 7 | 0 | 1 | 0.00 |  | 7 | 1 | 0 | 12.50 |  | 0 | 1 | 7 | 12.50 |  | 3 | 0 | 5 | 0.00 |
|  |  | Persistence | 11 | 8 | 0 | 3 | 27.27 |  | 10 | 1 | 0 | 0.00 |  | 0 | 0 | 11 | 100.00 |  | 0 | 1 | 10 | 90.91 |
|  |  |  |  |  |  |  |  |  |  |  |  |  |  |  |  |  |  |  |  |  |  |  |
| **Aggression** |  |  |  |  |  |  |  |  |  |  |  |  |  |  |  |  |  |  |  |  |  |  |
|  | **No behaviour** | Persistence | 17 | 13 | 3 | 1 | 76.47 |  | 12 | 4 | 1 | 70.59 |  | 9 | 4 | 4 | 52.94 |  | 12 | 4 | 1 | 70.59 |
|  |  | Incidence (low severity) | 9 | 7 | 1 | 1 | 11.11 |  | 6 | 2 | 1 | 22.22 |  | 3 | 1 | 1 | 20.00 |  | 6 | 2 | 1 | 22.22 |
|  |  | Incidence (high severity) | 1 | 0 | 1 | 0 | 0.00 |  | 1 | 0 | 0 | 0.00 |  | 0 | 0 | 1 | 100.00 |  | 0 | 0 | 1 | 100.00 |
|  |  |  |  |  |  |  |  |  |  |  |  |  |  |  |  |  |  |  |  |  |  |  |
|  | **Low severity** | Remission (no behaviour) | 7 | 5 | 2 | 0 | 71.43 |  | 3 | 4 | 0 | 42.86 |  | 0 | 4 | 3 | 0.00 |  | 4 | 3 | 0 | 57.14 |
|  |  | Persistence | 46 | 27 | 16 | 3 | 34.78 |  | 20 | 21 | 5 | 45.65 |  | 9 | 11 | 26 | 23.91 |  | 20 | 18 | 8 | 39.13 |
|  |  | Incidence (high severity) | 7 | 5 | 1 | 1 | 14.29 |  | 2 | 4 | 1 | 14.29 |  | 1 | 2 | 4 | 57.14 |  | 3 | 2 | 2 | 28.57 |
|  |  |  |  |  |  |  |  |  |  |  |  |  |  |  |  |  |  |  |  |  |  |  |
|  | **High severity** | Remission (no behaviour) | 2 | 1 | 1 | 0 | 0.00 |  | 1 | 0 | 1 | 50.00 |  | 0 | 1 | 1 | 0.00 |  | 1 | 0 | 1 | 50.00 |
|  |  | Remission (low severity) | 8 | 3 | 3 | 2 | 37.50 |  | 4 | 3 | 1 | 37.50 |  | 0 | 1 | 7 | 12.50 |  | 2 | 4 | 2 | 50.00 |
|  |  | Persistence | 24 | 10 | 10 | 4 | 16.67 |  | 3 | 15 | 6 | 25.00 |  | 1 | 2 | 21 | 87.50 |  | 6 | 7 | 11 | 45.83 |
| **Destruction of Property** |  |  |  |  |  |  |  |  |  |  |  |  |  |  |  |  |  |  |  |  |  |  |
|  | **No behaviour** | Persistence | 33 | 31 | 1 | 1 | 93.94 |  | 27 | 5 | 1 | 81.82 |  | 20 | 7 | 6 | 60.61 |  | 25 | 7 | 1 | 75.76 |
|  |  | Incidence (low severity) | 13 | 13 | 0 | 0 | 0.00 |  | 12 | 1 | 0 | 7.69 |  | 7 | 3 | 3 | 23.08 |  | 13 | 0 | 0 | 0.00 |
|  |  | Incidence (high severity) | 0 | 0 | 0 | 0 | NA |  | 0 | 0 | 0 | NA |  | 0 | 0 | 0 | NA |  | 0 | 0 | 0 | NA |
|  |  |  |  |  |  |  |  |  |  |  |  |  |  |  |  |  |  |  |  |  |  |  |
|  | **Low severity** | Remission (no behaviour) | 5 | 3 | 2 | 0 | 60.00 |  | 2 | 2 | 1 | 40.00 |  | 1 | 0 | 4 | 20.00 |  | 2 | 2 | 1 | 40.00 |
|  |  | Persistence | 38 | 34 | 2 | 2 | 5.26 |  | 18 | 11 | 9 | 28.95 |  | 6 | 8 | 24 | 21.05 |  | 23 | 6 | 9 | 15.79 |
|  |  | Incidence (high severity) | 8 | 7 | 0 | 1 | 12.50 |  | 5 | 3 | 0 | 0.00 |  | 2 | 3 | 3 | 37.50 |  | 6 | 1 | 1 | 12.50 |
|  |  |  |  |  |  |  |  |  |  |  |  |  |  |  |  |  |  |  |  |  |  |  |
|  | **High severity** | Remission (no behaviour) | 2 | 1 | 1 | 0 | 0.00 |  | 1 | 0 | 1 | 50.00 |  | 0 | 1 | 1 | 0.00 |  | 1 | 1 | 0 | 50.00 |
|  |  | Remission (low severity) | 9 | 7 | 1 | 1 | 11.11 |  | 6 | 1 | 2 | 11.11 |  | 0 | 6 | 3 | 66.67 |  | 5 | 2 | 2 | 22.22 |
|  |  | Persistence | 13 | 11 | 2 | 0 | 0.00 |  | 3 | 5 | 5 | 38.46 |  | 0 | 4 | 9 | 69.23 |  | 5 | 5 | 3 | 23.08 |
|  |  |  |  |  |  |  |  |  |  |  |  |  |  |  |  |  |  |  |  |  |  |  |
| **Any BtC** |  |  |  |  |  |  |  |  |  |  |  |  |  |  |  |  |  |  |  |  |  |  |
|  | **No behaviour** | Persistence | 11 | 8 | 3 | 0 | 72.73 |  | **7** | 3 | 1 | 63.64 |  | 6 | 2 | 2 | 60.00 |  | 8 | 2 | 1 | 72.73 |
|  |  | Incidence (low severity) | 6 | 5 | 0 | 1 | 0.00 |  | 2 | 3 | 1 | 50.00 |  | 1 | 4 | 1 | 66.67 |  | 4 | 1 | 1 | 16.67 |
|  |  | Incidence (high severity) | 0 | 0 | 0 | 0 | NA |  | 0 | 0 | 0 | NA |  | 0 | 0 | 0 | NA |  | 0 | 0 | 0 | NA |
|  |  |  |  |  |  |  |  |  |  |  |  |  |  |  |  |  |  |  |  |  |  |  |
|  | **Low severity** | Remission (no behaviour) | 6 | 3 | 3 | 0 | 50.00 |  | 2 | 4 | 0 | 33.33 |  | 2 | 0 | 4 | 33.33 |  | 3 | 2 | 1 | 50.00 |
|  |  | Persistence | 44 | 23 | 15 | 6 | 34.09 |  | 19 | 12 | 13 | 27.27 |  | 14 | 9 | 21 | 20.45 |  | 20 | 13 | 11 | 29.55 |
|  |  | Incidence (high severity) | 10 | 5 | 4 | 1 | 10.00 |  | 2 | 2 | 6 | 60.00 |  | 1 | 2 | 7 | 70.00 |  | 3 | 4 | 3 | 30.00 |
|  |  |  |  |  |  |  |  |  |  |  |  |  |  |  |  |  |  |  |  |  |  |  |
|  | **High severity** | Remission (no behaviour) | 1 | 1 | 0 | 0 | 0.00 |  | 1 | 0 | 0 | 100.00 |  | 0 | 0 | 1 | 0.00 |  | 1 | 0 | 0 | 100.00 |
|  |  | Remission (low severity) | 11 | 4 | 2 | 5 | 18.18 |  | 3 | 1 | 7 | 9.09 |  | 0 | 1 | 10 | 9.09 |  | 4 | 2 | 5 | 18.18 |
|  |  | Persistence | 32 | 6 | 15 | 11 | 34.38 |  | 2 | 7 | 23 | 71.88 |  | 1 | 1 | 30 | 93.75 |  | 6 | 7 | 19 | 59.38 |
|  |  |  |  |  |  |  |  |  |  |  |  |  |  |  |  |  |  |  |  |  |  |  |
| *Notes:* NA = Not Applicable, percentage could not be calculated due to no counts of data | | | | | | | | | | | | | | | | | | | | | | |

| **Supplementary Table 8.** External validation of prediction models showing percentage of correct classifications of incidence, persistence and remission of behaviour from Time 1 to Time 2 for presence of behaviour (no behaviour versus low and high behaviour composite group) and severity of behaviour (low behaviour versus high behaviour). | | | | | | | | | | |  |
| --- | --- | --- | --- | --- | --- | --- | --- | --- | --- | --- | --- |
|  |  | **Presence of behaviour % correct** | | | |  | **Level of behaviour % correct** | | | | |
|  |  | Persistence of no behaviour | Persistence behaviour^a^ | Incidence | Remission |  | Persistence of low behaviour | Persistence of high behaviour | Incidence | Remission |  |
| **Self-injurious behaviour** | KNN | 97.50 | 11.48 | 7.69 | 85.71 |  | 0.00 | 27.27 | 12.50 | 0.00 |  |
|  | Logistic regression | 97.50 | 6.56 | 15.38 | 100.00 |  | 2.94 | 0.00 | 0.00 | 12.50 |  |
|  | GMM | 50.00 | 85.25 | 76.92 | 42.86 |  | 2.94 | 100.00 | 87.50 | 12.50 |  |
|  | Random Forest | 67.50 | 62.30 | 53.85 | 71.43 |  | 5.88 | 90.91 | 75.00 | 0.00 |  |
| **Aggression** | KNN | 76.47 | 47.06 | 30.00 | 66.67 |  | 34.78 | 16.67 | 14.29 | 37.50 |  |
|  | Logistic regression | 70.59 | 65.88 | 30.00 | 44.44 |  | 45.65 | 25.00 | 14.29 | 37.50 |  |
|  | GMM | 52.94 | 87.06 | 50.00 | 0.00 |  | 23.91 | 87.50 | 57.14 | 12.50 |  |
|  | Random Forest | 70.59 | 63.53 | 40.00 | 55.56 |  | 39.13 | 45.83 | 28.57 | 50.00 |  |
| **Destruction of property** | KNN | 93.94 | 13.24 | 0.00 | 57.14 |  | 5.26 | 0.00 | 12.50 | 11.11 |  |
|  | Logistic regression | 81.82 | 52.94 | 7.69 | 42.86 |  | 28.95 | 38.46 | 0.00 | 11.11 |  |
|  | GMM | 60.61 | 88.24 | 46.15 | 14.29 |  | 21.05 | 69.23 | 37.50 | 66.67 |  |
|  | Random Forest | 75.76 | 42.65 | 0.00 | 42.86 |  | 15.79 | 23.08 | 12.50 | 22.22 |  |
| **Any BtC** | KNN | 72.73 | 60.82 | 16.67 | 57.14 |  | 34.09 | 34.38 | 10.00 | 18.18 |  |
|  | Logistic regression | 63.64 | 73.20 | 66.67 | 42.86 |  | 27.27 | 71.88 | 60.00 | 9.09 |  |
|  | GMM | 60.00 | 83.51 | 83.33 | 28.57 |  | 20.45 | 93.75 | 70.00 | 9.09 |  |
|  | Random Forest | 72.73 | 65.98 | 33.33 | 57.14 |  | 29.55 | 59.38 | 30.00 | 18.18 |  |
| ^a^low or high severity behaviour | | | | | | | | | | |  |

| **Supplementary Table 9.** Coefficients and significance of predictor variables for multinominal logistic regression models of self-injurious behaviour, aggression, destruction of property and any BtC | | | | | | | | | |
| --- | --- | --- | --- | --- | --- | --- | --- | --- | --- |
|  | **Low severity behaviour^a^** | | | |  | **High severity behaviour^a^** | | | |
|  | B | SE | *p*-value | 95% CI |  | B | SE | *p*-value | 95% CI |
| **Self-Injurious behaviour** | | | | | | | | | |
| Constant | -3.104 | .367 | **<.001**** | -3.824, |  | -5.723 | .636 | **<.001**** | -6.970, -4.477 |
| Age | .085 | .028 | **.003*** | .030, .140 |  | .118 | .040 | .**003*** | .040, .197 |
| Gender | -.262 | .237 | .269 | -.726, .203 |  | -.326 | .347 | .348 | -1.006, .354 |
| Adaptive behaviour | .402 | .232 | .084 | -.054, .857 |  | .841 | .362 | .**020*** | .130, 1.551 |
| Autism | -.062 | .202 | .760 | -.458, .335 |  | -.383 | .295 | .195 | -.961, .196 |
| Repetitive behaviour | .086 | .067 | .197 | -.045, .217 |  | .094 | .092 | .307 | -.086, .274 |
| Impulsivity | .126 | .051 | **.013*** | .026, .227 |  | .307 | .080 | **<.001**** | .150, .464 |
| Overactivity | .116 | .055 | **.034*** | .009, .223 |  | .193 | .077 | **.012*** | .042, .344 |
| **Aggression** | | | | | | | | | |
| Constant | -2.138 | .328 | **<.001**** | -2.780, -1.496 |  | -6.412 | .670 | **<.001**** | -7.724, -5.100 |
| Age | .075 | .027 | **.005*** | .023, .128 |  | .100 | .041 | **.015*** | .019, .180 |
| Gender | -.668 | .213 | **.002*** | -1.085, -.251 |  | -.879 | .351 | **.012*** | -1.568, -.190 |
| Adaptive behaviour | .625 | .213 | **.003*** | .207, 1.043 |  | 1.073 | .345 | **.002*** | .396, 1.749 |
| Autism | .041 | .189 | .828 | -.330, .413 |  | -.052 | .292 | .858 | -.624, .520 |
| Health problems | .116 | .083 | .163 | -.047, .279 |  | .324 | .113 | **.004*** | .102, .547 |
| Repetitive behaviour | -.076 | .076 | .317 | -.224, .073 |  | -.112 | .108 | .303 | -.324, .101 |
| Obsessive behaviour | .087 | .077 | .261 | -.064, .237 |  | .156 | .117 | .179 | -.072, .385 |
| Impulsivity | .326 | .053 | **<.001**** | .223, .429 |  | .624 | .089 | **<.001**** | .450, .798 |
| Overactivity | .024 | .054 | .656 | -.082, .130 |  | .163 | .077 | **.033*** | .013, .313 |
| **Destruction of Property** | | | | | | | | | |
| Constant | -3.478 | .378 | **<.001**** | -4.219, -2.738 |  | -6.914 | .728 | **<.001**** | -8.340, -5.488 |
| Age | .092 | .028 | **.001*** | .038, .147 |  | .121 | .042 | **.004*** | .039, .203 |
| Gender | -.163 | .229 | .477 | -.611, .285 |  | -.635 | .377 | .092 | -1.373, .104 |
| Adaptive behaviour | .532 | .231 | **.021*** | .079, .984 |  | 1.014 | .362 | **.005*** | .305, 1.724 |
| Autism | .237 | .199 | .234 | -.153, .627 |  | .260 | .299 | .384 | -.325, .846 |
| Health problems | .155 | .083 | .060 | -.007, .317 |  | .311 | .112 | **.005*** | .092, .530 |
| Repetitive behaviour | -.088 | .077 | .252 | -.238, .062 |  | -.148 | .109 | .174 | -.361, .065 |
| Obsessive behaviour | .023 | .079 | .770 | -.132, .179 |  | .006 | .118 | .959 | -.225, .237 |
| Impulsivity | .315 | .052 | **<.001**** | .212, .417 |  | .470 | .092 | **<.001**** | .291, .649 |
| Overactivity | .093 | .052 | .076 | -.010, .196 |  | .304 | .080 | **<.001**** | .147, .460 |
| **Any BtC** | | | | | | | | | |
| Constant | -1.959 | .336 | **<.001**** | -2.618, -1.300 |  | -5.935 | .579 | **<.001**** | -7.069, -4.802 |
| Age | .081 | .028 | **.005*** | .025, .136 |  | .142 | .039 | **<.001**** | .066, .217 |
| Gender | -.675 | .216 | **.002*** | -1.098, -.251 |  | -.840 | .320 | **.009*** | -1.466, -.214 |
| Adaptive behaviour | .677 | .219 | **.002*** | .247, 1.107 |  | 1.248 | .321 | **<.001**** | .619, 1.877 |
| Autism | -.077 | .198 | .699 | -.465, .312 |  | -.228 | .274 | .404 | -.764, .308 |
| Health problems | .114 | .089 | .198 | -.059, .288 |  | .399 | .111 | **<.001**** | .181, .617 |
| Repetitive behaviour | .013 | .079 | .868 | -.142, .168 |  | -.030 | .102 | .770 | -.231, .171 |
| Obsessive behaviour | -.017 | .080 | .831 | -.175, .140 |  | .068 | .108 | .530 | -.144, .280 |
| Impulsivity | .346 | .056 | **<.001**** | .237, .455 |  | .599 | .078 | **<.001**** | .446, .751 |
| Overactivity | .075 | .058 | .196 | -.039, .189 |  | .216 | .074 | **.003*** | .072, .360 |
| ^a^Reference category: No behaviour  SE = standard error; CI = confidence intervals  **p*<.05; ***p*<001 | | | | | | | | | |

| **Self-injurious behaviour** | **Aggression** |
| --- | --- |
|  |  |
| **Destruction of property** | **Any BtC** |
|  |  |
| **Supplementary Figure 1.** Relative importance (mean decrease in gini impurity) of predictor variables and 95% confidence intervals for random forest models of self-injurious behaviour, aggression, destruction of property and any BtC. | |
